# A Practical Method for No-Reflow Treatment

**DOI:** 10.1155/2016/9596123

**Published:** 2016-02-11

**Authors:** Mustafa Cetin, Emrullah Kiziltunc, Zehra Güven Cetin, Harun Kundi, Birsen Gulkan, Hülya Cicekcioglu

**Affiliations:** ^1^Cardiology Department, Numune Education and Research Hospital, Cardiology Department, 06100 Ankara, Turkey; ^2^Cardiology Department, Dr. Nafiz Korez State Hospital, 06100 Ankara, Turkey

## Abstract

No-reflow is an undesirable result of percutaneous coronary interventions. Vasoactive drug administration at the distal part of the coronary artery is suggested as a therapeutic option for no-reflow treatment. Here, we represent two cases of successful no-reflow management with previously used monorail balloon at the same procedure as a hand-made distal infusion catheter.

## 1. Introduction

No-reflow is an undesirable result of percutaneous coronary interventions (PCI) [1]. Intracoronary (IC) vasodilators such as verapamil, nitroprusside, or adenosine are being administered for the treatment of no-reflow via the guiding catheter [2], but sometimes distal flow restoration is not satisfactory especially in patients with TIMI 0 flow. Vasoactive drug administration at the distal part of the coronary artery is suggested as a treatment option for no-reflow and some distal infusion catheters [3] and over-the-wire (OTW) balloon catheters [4] are being used for this purpose. However, OTW catheters need long guide wires and changing a short wire with a long wire has the risk of wire loss. Distal infusion catheters are special catheters for drug infusion at various vascular sites, but these catheters are not always available. Monorail balloon catheters are the most widely used catheters in routine clinical practice. Here, we describe two cases of successful no-reflow management by previously used monorail balloon at the same procedure as a hand-made distal infusion catheter.

## 2. Case 1

**Figure 1:**
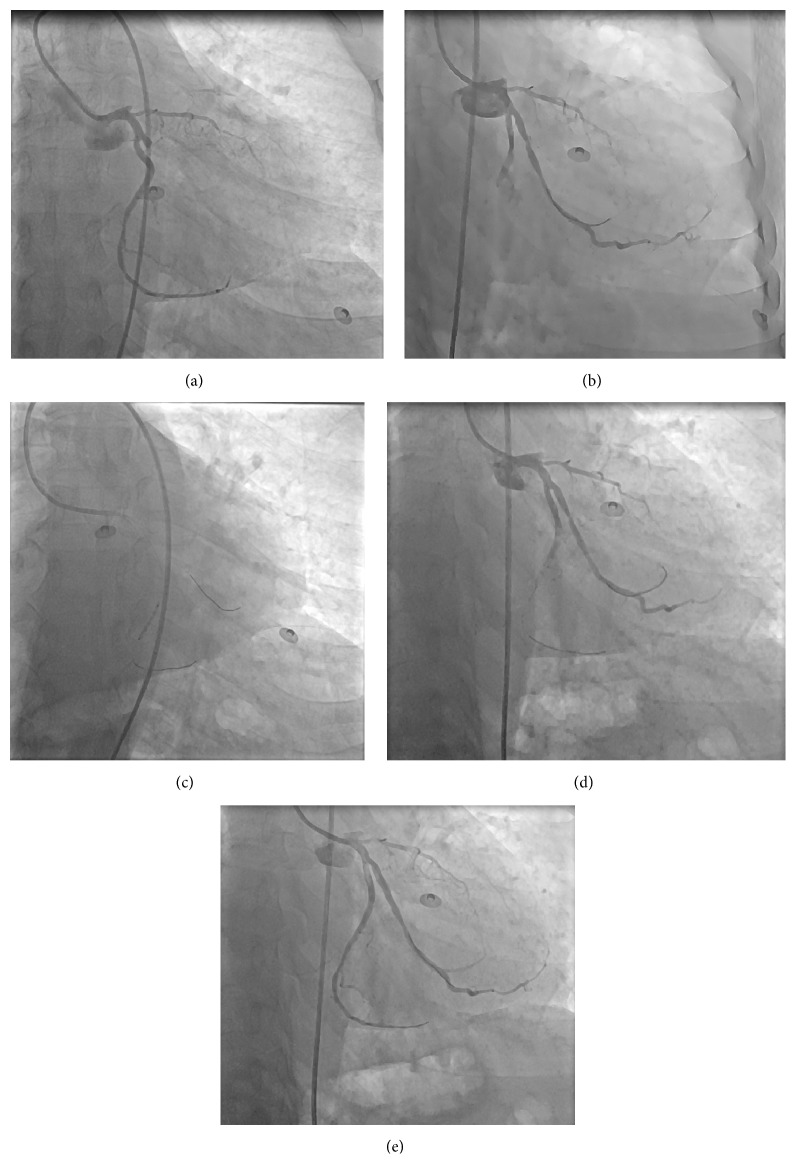
(a) The first angiogram of the patient. (b) After balloon dilatation to the first OM, Cx midportion was totally occluded due to thrombus shift. (c) A 1.5/20 mm monorail balloon was dilated at the Cx occlusion. (d) No-reflow developed. (e) 1.5/20 mm monorail balloon was perforated and was inserted to the distal part of the Cx; afterwards 250 mcg adenosine was injected via the balloon. Successful distal flow was restored. OM: obtuse marginalis, Cx: circumflex artery.

**Figure 2:**
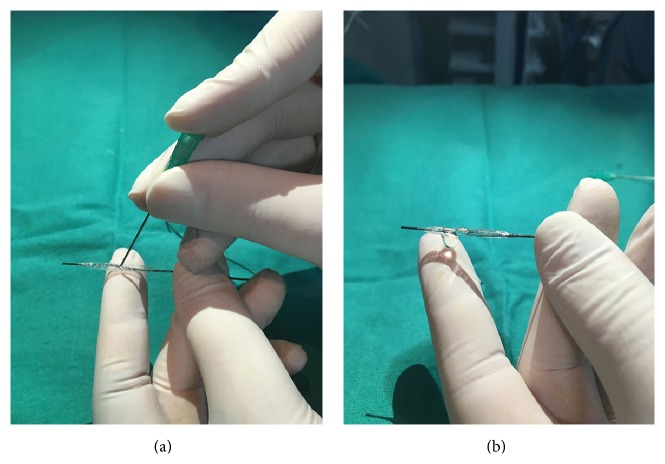
Preparation of balloon before distal infusion. (a) First the balloon is filled with saline and perforated with a needle from perpendicular four different sites. It is important to not cross the opposite layer of the balloon. (b) The perforated balloon is flushed from the hub with adenosine solution and the bubbles are removed.

A 50-year-old male patient was taken to catheterization laboratory at the third hour of his pain with the diagnosis of acute posterolateral myocardial infarction (MI). The patient was pretreated with acetylsalicylic acid 300 mg, prasugrel 60 mg, and unfractionated heparin 100 mg/kg. Infarct related artery was first obtuse marginalis (OM) (Figure 1(a)). Total occlusion was crossed with a 0.014 inch floppy wire and the lesion was predilated with a 2.5/20 mm monorail balloon at 12 atm pressure. Control angiography revealed a thrombus shift to circumflex artery (Cx) which ceased the distal flow (Figure 1(b)). The occlusion was crossed with a second 0.014-inch floppy wire and the occlusion site was dilated with a 1.5/20 mm monorail balloon at 16 atm pressure (Figure 1(c)). After balloon angioplasty no-reflow developed. Intracoronary adenosine (250 mcg) was administered, but distal flow could not be restored (Figure 1(d)). Therefore we decided to give adenosine at the distal part of Cx and the previously used 1.5/20 mm monorail balloon was retrieved and perforated with a needle at four different sites. The perforated balloon was flushed from the hub with adenosine solution (1 cc/100 mcg) and bubbles were removed (Figures 2(a) and 2(b)). Then it was inserted to the distal part of the Cx and 250 mcg adenosine was injected via the balloon. Control angiography revealed TIMI 3 flow (Figure 1(e)). First OM was stented with a 3.5/12 mm bare metal stent and the procedure was terminated without any other complication.

## 3. Case 2

**Figure 3:**
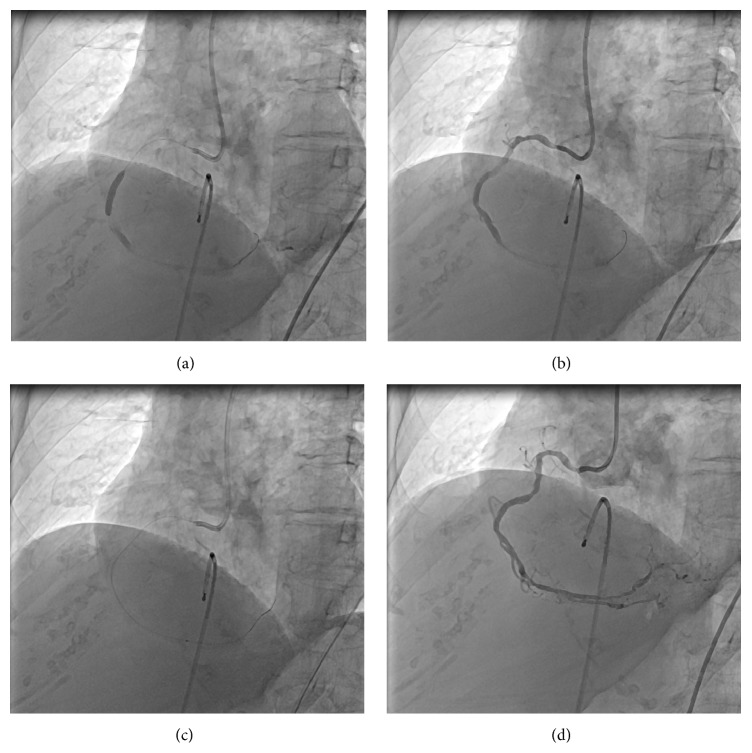
(a) 3.0/18 mm drug eluting stent deployment. (b) After stent deployment no-reflow developed. (c) 250 mcg adenosine was injected via the previously used perforated 1.20/12 mm monorail balloon to the distal part of the RCA. (d) TIMI 3 distal flow was restored. RCA: right coronary artery, TIMI: thrombolysis in myocardial infarction.

An 87-year-old female patient admitted with acute inferior MI and complete AV block at the thirteenth hour of her symptoms. The patient was taken to catheterization laboratory for primary PCI. She was pretreated with acetylsalicylic acid 300 mg, ticagrelor 180 mg, and 85 mg/kg unfractionated heparin. Right coronary artery (RCA) was totally occluded at the midportion. A 0.014-inch floppy wire was inserted to the distal part of the RCA. The lesion was predilated with 1.20/12 mm and 3.0/15 mm monorail balloons, respectively, and a 3.0/18 mm drug eluting stent was implanted at 16 atm (Figure 3(a)). No-reflow developed after stent deployment. We administered 250 mcg adenosine via the guiding catheter but could not achieve any distal flow (Figure 3(b)). Afterwards, previously used 1.20/12 mm monorail balloon was perforated and flushed with adenosine solution (1 cc/100 mcg) and bubbles were removed. Then it was inserted to the distal part of the RCA and 250 mcg adenosine was injected via the balloon (Figure 3(c)). TIMI 3 flow was restored (Figure 3(d)).

## 4. Discussion

No-reflow increases mortality and hospital stay in acute MI patients [5]. Distal microembolisation, endothelial dysfunction, and reperfusion injury (by increasing the microvascular resistance) are the proposed mechanisms of no-reflow [6]. Antiplatelet agents, thrombus aspiration, distal embolic protection devices, and vasodilator drugs can be used for prevention and treatment of no-reflow [7]. IC vasodilator administration through the guiding catheter is frequently performed to resolve no-reflow. But it is obvious that administered vasodilator agents cannot penetrate to the coronary microcirculation especially in patients with TIMI 0 flow because there is no blood flow at the distal part of the vessel during no-reflow period. In addition, when vasodilator drug is administered by the guiding catheter, it penetrates to other coronary territories and the vasodilator drug concentration dilutes.

Administration of the vasodilator drugs at the distal part of the coronary can deal with these problems and vasoactive drug utility can be increased at the distal part of the coronary artery. OTW balloon catheters [8] and distal infusion catheters [9, 10] have been using for this purpose and positive results were reported with distal vasodilator drug injection. However these two types of catheters have some disadvantages. Distal infusion catheters (like ClearWay, Atrium Medical Corporation, Hudson, NH, USA/multifunction probing catheter, Boston Scientific Corporation, Boston, MA) are suitable for drug administration at the distal part of coronary artery, but these catheters are not routinely found in most of the laboratories. OTW balloons are not the first choices in daily practice and need a long guidewire. Wire exchange for OTW balloon usage has the risk of wire loss and failure of rewiring, a very annoying situation for the operator. Monorail balloons are frequently used in daily practice. They are easily retrievable and they can be used with short guidewires. Here, we described successful usage of a monorail balloon for distal vasodilator drug infusion. There may be some safety concerns about inserting a perforated balloon to the distal part of the coronary and drug infusion through this balloon, but we did not encounter any complication with this method and reached successful results. Flushing the perforated balloon from the hub takes away the risk of air bubble embolism. Perforating the balloon form multiple sites reduces the risk of coronary dissection due to saline flow because injected saline's pressure is reduced by multiple holes. In addition, this method does not impose an additional cost to the procedure. Health expenditure is very important all around the world. We think that distal drug infusion with this method is safe and effective at least like other catheters; therefore using a new catheter and rising the cost of the procedure are unnecessary. In conclusion, perforation of a monorail balloon with a needle and using as a distal infusion catheter can be used for no-reflow treatment. We achieved favorable results with this off-label usage. This method is always available, easy, and safe and has no additional cost.
